# Impact of Time-Restricted Feeding on Adaptation to a 6-Hour Delay Phase Shift or a 12-Hour Phase Shift in Mice

**DOI:** 10.3390/nu14153025

**Published:** 2022-07-23

**Authors:** Baoyin Ren, Yingzhi Huang, Jiayang Zhang, Jiazhi Li, Zhaiyi Liu, Youfei Guan, Lihong Chen, Guangrui Yang

**Affiliations:** 1 School of Bioengineering, Dalian University of Technology, Dalian 116024, China; baoyinren@mail.dlut.edu.cn (B.R.); zhaiyi31@163.com (Z.L.); 2 Advanced Institute for Medical Sciences, Dalian Medical University, Dalian 116024, China; hyz1398034902@163.com (Y.H.); zhangjiayang0912@163.com (J.Z.); ydshangan2021@163.com (J.L.); guanyf@dmu.edu.cn (Y.G.); lihong@dmu.edu.cn (L.C.)

**Keywords:** adaptation, behavior, phase shift, sepsis, time-restricted feeding

## Abstract

Nowadays, more and more people are suffering from circadian disruption. However, there is no well-accepted treatment. Recently, time-restricted feeding (TRF) was proposed as a potential non-drug intervention to alleviate jet lag in mice, especially in mice treated with a 6-h advanced phase shift. Here, we challenged C57BL/6 mice with a 6-h delay phase shift or a 12-h shift (day-night reversal) combined with 6- or 12-h TRF within the dark phase and found the beneficial effects of given TRF strategies in certain phase-shifting situations. Although behavioral fitness did not correlate well with health status, none of the TRF strategies we used deteriorated lipopolysaccharide-induced sepsis. These findings improve our understanding of the benefits of TRF for adaptation to circadian disruption.

## 1. Introduction

In modern society, more and more people undergo frequent circadian disruption due to shift working, artificial light at night, air flight crossing time zones, social activities, overuse of smartphones or tablets, etc. However, despite many drugs or chemicals targeting molecular clocks, there is no well-accepted medical treatment for circadian rhythm disorders [1,2]. Recently, we proposed the possibility of reducing jet lag with the timing of meals, or time-restricted feeding (TRF), as a non-drug intervention [3].

On the one hand, circadian rhythms determine the times when food should be consumed, which are most often coincident with active periods [4]. On the other hand, dietary patterns can affect circadian rhythms, and rhythmic feeding behavior, as the most potent non-photic zeitgeber, is important for health when aligned with light cues [5,6]. In mice, for instance, restricting food availability to the active phase (dark phase) can prevent metabolic syndrome [7] and reverse preexisting obesity and impaired glucose tolerance caused by high-fat diets [8]. The effective benefits, however, are lost when feeding is restored to an ad libitum pattern whose rhythm is relatively unstable [8].

In addition to the benefit under regular light/dark conditions, TRF has received some attention in the studies of rodent models under disrupted schedules, although it has not yet been established as a strategy for alleviating circadian disorders. For example, the speed of re-entrainment was accelerated in rats treated with a 6-h (6-h) advanced shift by feeding scheduled to the first two hours during the dark phase [9,10]. Another study showed that mice were able to adjust quickly to weekly repeated 12-h shifts (day-night reversal) by restricting food intake to the dark phase [11]. Despite this, none of these studies investigated whether their TRF strategies positively impacted health that might be compromised by disturbed circadian rhythms.

To test if mice with faster adaptation are healthier, we recently applied various 6-h TRF strategies on mice treated with a 6-h advanced shift of light/dark cycle, the most frequently used phase shift in chronobiology, as the first attempt to study the effect of TRF on both behavior and health [3]. The results showed that feeding scheduled to late night after the shift facilitated behavioral adaptation, as well as improved the survival of septic mice that were induced by lipopolysaccharide (LPS), raising the feasibility of meal timing to alleviate the circadian clock disorders. Here, to expand our understanding of the benefits of TRF on jet-lagged mice, we applied a 6-h delay phase shift or a 12-h phase shift (day-night reversal) schedule to determine whether any TRF strategies would be helpful in adapting to jet lag and improving physical health.

## 2. Materials and Methods

### 2.1. Mice

Eight- to twelve-week-old male C57BL/6 mice were individually housed in ventilated and light-proof cabinets (Probecare, Wuhan, China) under 12-h light:12-h dark condition (LD). Before experiments, all mice had free access to water and a normal chow diet (Changsheng Biotechnology, Benxi, China). The intensity of light at the animal level during the day was about 100 lux. Light-on was defined as zeitgeber time 0 (ZT0), and light-off, ZT12. The ambient temperature was 24 ± 1 °C.

### 2.2. Schedules

**Figure 1:**
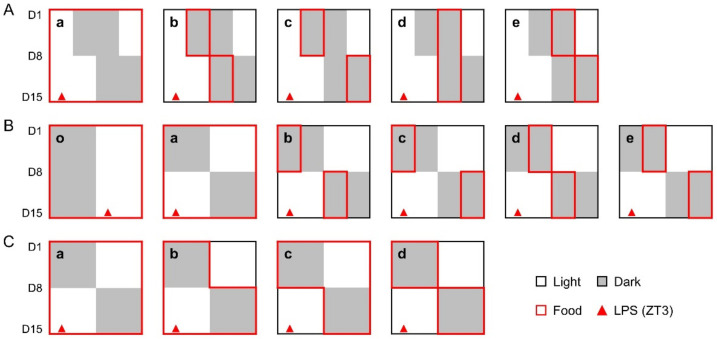
Study design. (**A**), The mice in experiment 1 were treated with a 6-h delayed phase shift, combined with or without 6-h TRFs; (**B**), The mice in experiment 2 were treated with a 12-h phase shift, combined with or without 6-h TRFs; (**C**), The mice in experiment 3 were treated with a 12-h phase shift, combined with or without 12-h TRFs. One week after the relevant shift, mice in all groups were treated with LPS (15 mg/kg body weight, ip) at ZT3. Health status was monitored for 7 days.

The study contains three experiments with different schedules in a combination of phase shifts and TRF strategies (Figure 1). (1) All mice were subjected to a 6-h delayed phase shift with or without TRFs. Group a, the control group, has free access to food throughout the study. The mice in other groups were given food in the first (ZT12-18) or the second half (ZT18-ZT24) of the night one week prior to and following the shift, as indicated in Figure 1A. (2) Mice were divided into 6 groups. Group o was maintained under regular light/dark cycles with free access to food. All other groups were treated with a 12-h phase shift, combined with or without TRFs, as indicated in Figure 1B. (3) All mice were treated with a day-night reversal. Group a, the control group, has free access to food throughout the study. The mice in other groups were subjected to food deprivation during the light phase one week before (group b), after (group c), or both before and after (group d) the day-night reversal (Figure 1C). One week after the relevant shift, the mice in all groups were peritoneally injected with LPS (Solarbio, Beijing, China) at ZT3 to induce sepsis [3].

### 2.3. Wheel Running Activity

Mice were individually housed in a running wheel-equipped cage (Probecare, Wuhan, China), and wheel rotation was recorded throughout the experiments. ClockLab software (Actimetrics, Wilimette, IL, USA) was used for data collection and analysis. The number of days until stable re-entrainment after the shift was calculated as previously described [3,12] with modification. Briefly, entrainment to the new light/dark schedule was defined as stable activity onset or offset, whichever occurred later, for four consecutive days or days before LPS treatment if less than four.

### 2.4. Sepsis Model

Sepsis was induced, and health status was evaluated as previously described [3].

### 2.5. Statistical Analysis

Data were analyzed using Prism 8 (GraphPad Software, Inc., San Diego, CA, USA). One-way ANOVA (nonparametric or mixed) with Tukey’s test was used in multiple groups. The log-rank test was used to compare survival distributions. A Chi-square test was used for the analysis of the numbers of active and inactive mice after LPS treatment. Data correlations between septic scores and activities were evaluated using Spearman’s correlation analysis. For statistical purposes, we defined active as the number of total wheel revolutions for 7 days right after LPS treatment as more than 5000. All data were expressed as mean ± SEM. All statistical tests were two-sided.

## 3. Results

### 3.1. Effect of 6-h TRFs on Mice Treated with a 6-h Delay Shift

**Figure 2:**
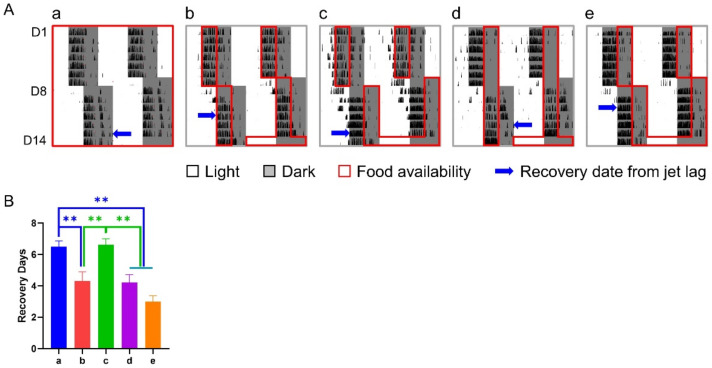
Effect of 6-h TRFs on behavioral adaptation in mice treated with a 6-h delay phase shift. (**A**) Representative double-plotted actograms of wheel running activity of mice in group a–e. Right and left blue arrows indicate the date when the mouse was re-entrained based on the calculation of onset and offset of daily activity, respectively. (**B**) Days for recovery from the shift. ** *p* < 0.01, one-way ANOVA with Tukey’s multiple comparisons test (*n* = 13–14).

Under the treatment of a 6-h delay shift combined with various 6-h TRFs, mice in groups b, d, and e adjusted significantly faster than those in groups a (control) and c, who recovered from the shift in 6.50 ± 0.36 and 6.62 ± 0.38 days, respectively, while groups b, d, and e took averagely 3–4 days to adapt to the shift (Figure 2).

To test if fast responders are healthier, we injected mice with LPS and evaluated their health status 3, 9, and 15 h after the treatment. Overall, the septic score of the levels of consciousness and responses to stimuli in all mice was gradually increased throughout these time points (Table 1). At the same time, the control group was the most severe, especially when compared with groups d and e at time points 9 and 15 and with group c at time point 15. No obvious difference was observed between the control group and group b.

**Figure 3:**
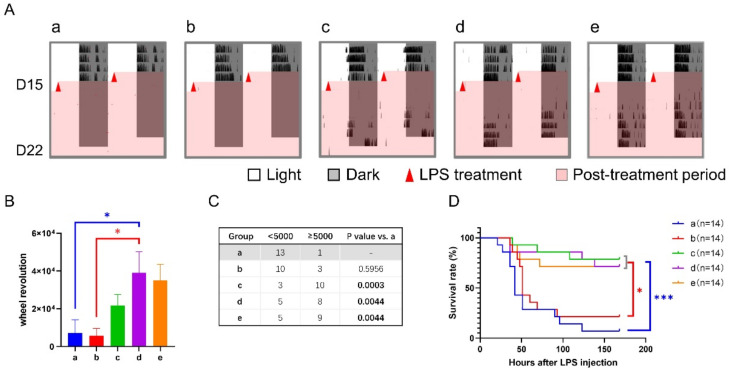
Effect of 6-h TRFs on behavior and health in mice treated with a 6-h delay phase shift and LPS injection. (**A**) Representative double-plotted actograms of wheel running activity after LPS treatment. (**B**) Total number of wheel revolutions after LPS injection. * *p* < 0.05, one-way ANOVA with Tukey’s multiple comparisons test. (**C**) The numbers of active and inactive mice after LPS treatment, Chi-square (and Fisher’s exact) test. (**D**) Effect of TRF on the survival of septic mice. *** *p* < 0.001, * *p* < 0.05, Log-rank test.

During sepsis, wheel revolution was continuously recorded. Groups a and b had little activity over the 7-d checking period, while groups c, d, and e recovered to a certain extent after a disturbance by LPS treatment (Figure 3A–C). Meanwhile, we monitored fatal events and found that the survival rates of groups c, d, and e were all more than 70% seven days after LPS treatment (Figure 3D). By contrast, only 7% in group a (*p* < 0.001 vs. group c, d, or e) and 21% in group b (*p* < 0.05 vs. group c, d, or e) survived the sepsis. These changes were consistent with their sepsis scores but not behavior.

### 3.2. Effect of 6-h TRFs on Mice Treated with a 12-h Shift

**Figure 4:**
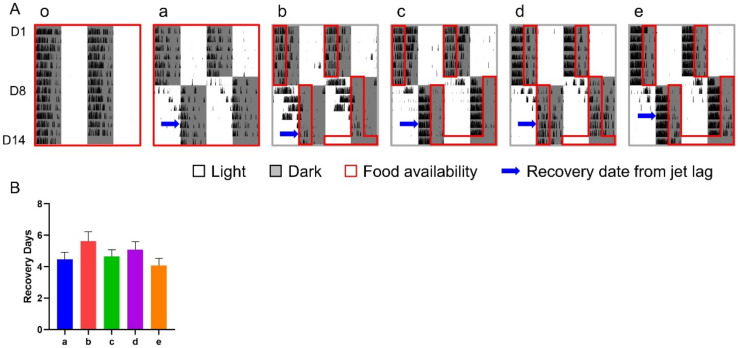
Effect of 6-h TRFs on behavioral adaptation in mice treated with a 12-h shift. (**A**), Representative double-plotted actograms of wheel running activity of mice in group o–e; (**B**), Days for recovery from the shift. None of the groups are statistically different from each other. One-way ANOVA with Tukey’s multiple comparisons test (*n* = 8–14).

Under the treatment of a 12-h shift combined with various 6-h TRFs, the number of days for re-entrainment ranged from 4.07 (group e) to 5.63 (group b) without obvious difference between groups (Figure 4).

However, the mice in different groups had different responses to LPS treatment when checked 9 and 15 h after LPS injection (Table 1). At time point 9, the level of consciousness in groups c and e was significantly lower than that in both control groups, i.e., groups o (no shift, no TRF) and a (no TRF). The response to stimuli revealed that all TRF groups had lower septic scores than the control groups. Similar patterns in the level of consciousness and response to stimuli were also seen at time point 15, suggesting TRF groups were healthier without distinctions among our TRF strategies.

**Figure 5:**
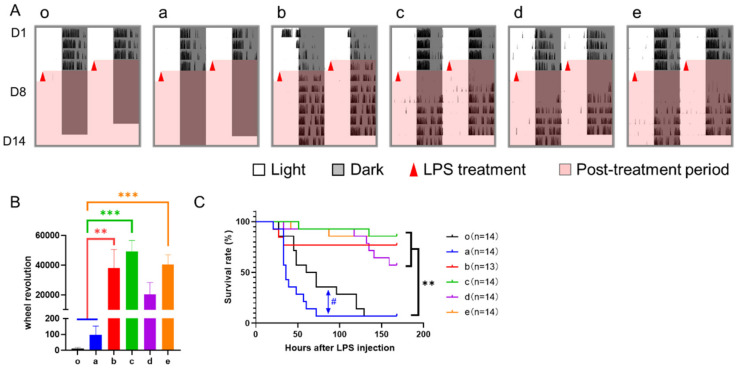
Effect of 6-h TRFs on behavior and health in mice treated with a 12-h phase shift and LPS injection. (**A**) Representative double-plotted actograms of wheel running activity after LPS treatment; (**B**) Total number of wheel revolutions after LPS injection. *** *p* < 0.001; ** *p* < 0.01, one-way ANOVA with Tukey’s multiple comparisons test. (**C**) Effect of TRF on survival of septic mice. ** *p* < 0.01; # *p* < 0.1, Log-rank test.

The results of wheel running activity after LPS treatment showed that both control groups had little activity throughout the 7 days of recording, while TRF groups, especially groups b, c, and e, recovered to a certain level after a transient disturbance by LPS treatment (Figure 5A,B). Consistently, the survival rates of septic mice in all TRF groups were much higher than those in controls (Figure 5C). In addition, despite the same death rate of the two control groups at the endpoint of the experiment, their survival curves separated from 2 to 5 days after LPS treatment with a decreased trend of survival rate in group a (*p* = 0.091).

### 3.3. Effect of 12-h TRFs on Mice Treated with a 12-h Shift

**Figure 6:**
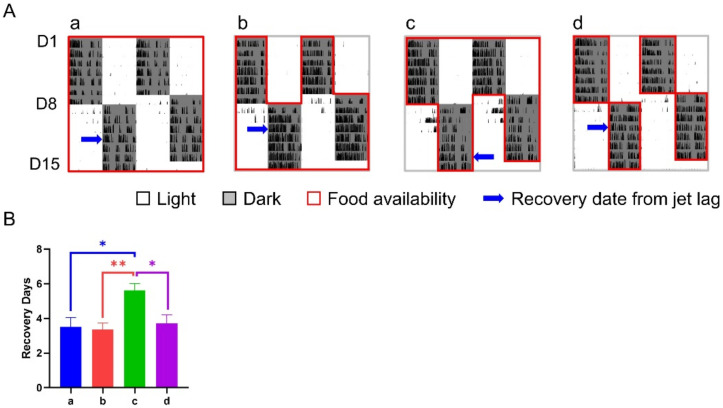
Effect of 12-h TRFs on behavioral adaptation in mice treated with a 12-h shift. (**A**) Representative double-plotted actograms of wheel running activity of mice in groups a–d. (**B**) Days for recovery from the shift. ** *p* < 0.01; * *p* < 0.05, one-way ANOVA with Tukey’s multiple comparisons test (*n* = 13–14).

Under the treatment of a 12-h shift combined with or without whole night TRFs, mice in group c (TRF applied after the shift only) took 5.62 ± 0.40 days for re-entrainment, which was roughly 2 days longer than any other group, including the controls (Figure 6).

However, the health status reflected by LPS-induced sepsis was inconsistent with their adaptability to the 12-h shift. The sepsis scores after 3 and 9 h of LPS treatment showed no statistical difference between groups (Table 1). At time point 15, group d had a lower septic score than groups a and b, while the mice in group c, the slower responders, were not statistically different from groups a, b, or d.

**Figure 7:**
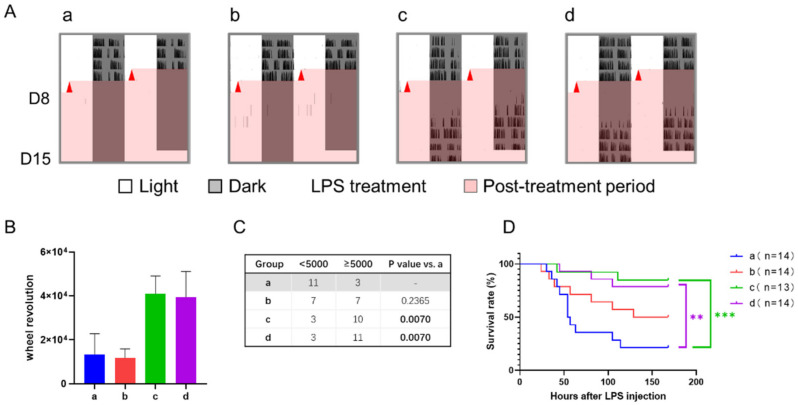
Effect of 12-h TRFs on behavior and health in mice treated with a 12-h delay phase shift and LPS injection. (**A**) Representative double-plotted actograms of wheel running activity after LPS treatment. (**B**) Total number of wheel revolutions after LPS injection. None of the groups were statistically different from each other. One-way ANOVA with Tukey’s multiple comparisons test. (**C**) The numbers of active and inactive mice after LPS treatment, Chi-square (and Fisher’s exact) test. (**D**) Effect of TRF on survival of septic mice. *** *p* < 0.001; ** *p* < 0.01, Log-rank test.

The results of wheel running activity revealed that the control group had little activity after LPS treatment, while other groups recovered to a certain level a few days later (Figure 7A). Although one-way ANOVA analysis showed that all groups had no statistical difference in wheel revolution between each other (Figure 7B), the number of active and inactive mice differed significantly among groups (Figure 7C). In group a, 21% of mice (3/14) were relatively active, while about 80% in groups c and d were active (*p* < 0.01). Group b was between them without statistical difference. Consistently, the survival rate of septic mice in groups c and d were significantly higher than that of the control group, and group b was in the middle (Figure 7D).

### 3.4. Correlation between Septic Score and Wheel-Running Activity

**Figure 8:**
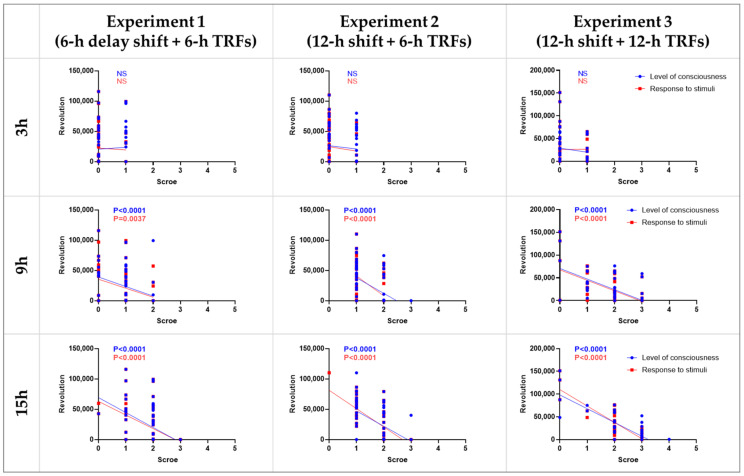
Correlation between septic scores and wheel revolutions. The X-axis in each graph represents the scores of the level of consciousness (blue) and response to stimuli (red) at 3, 9, and 15 h after LPS injection. The Y-axis represents the total number of wheel revolutions 7 days following LPS injection. NS, no statistical difference, Spearman correlation analysis.

Correlation between the septic scores at 3-, 9-, and 15-h and the number of 7-day wheel revolutions after LPS treatment in each experiment were calculated. The results showed that the scores at time points 9 and 15, but not 3, were negatively correlated with wheel revolutions (Figure 8). In addition, no significant difference was observed between the two score systems, i.e., the level of consciousness and response to stimuli.

## 4. Discussion

It has long been known that disruption of the biological clock results in adverse effects in many aspects in humans and mouse models [13]. However, there are no putative medications or guidelines yet. In recent years, TRF has drawn much attention in the field of circadian biology due to growing knowledge about its beneficial effects on health [5,7,8,14,15,16], while most of those studies were performed under normal light/dark conditions rather than disrupted schedules. We recently reported that food restricted to late night after a 6-h advanced phase shift accelerates re-entrainment with improved resilience to sepsis in mice, raising a feasible way to reduce jet lag [3]. To expand our knowledge, here we further investigated the effects of various 6-h or 12-h TRF strategies on a 6-h delay phase shift or a 12-h shift.

First, we treated C57BL/6 mice with a combination of a 6-h delayed phase shift and 6-h TRFs before and after the shift and found that group c was the only TRF group without an effect of accelerating adjustment. This might be due to the fact that TRF schedules in this group are 6 h apart before and after the shift, whereas all other groups did not have such a gap and therefore did not need to spend much time to adjust. However, the LPS sepsis model revealed that not all faster responders were healthier (group b) and vice versa (group c). Among the three healthier groups (c, d, and e), groups c and e were treated with late-night TRF after the shift, which is consistent with our previous report on mice treated with a 6-h advanced phase shift [3]. For group d, a possible explanation is that TRFs before and after shifts lie in the same real-time window, which may minimize the changes in endogenous clocks, and thus offer benefits.

Then we investigated the effect of 6-h TRF on a 12-h shift, i.e., day-night reversal, and found that any 6-h TRF strategy had a significant beneficial effect on health, while there was no difference in the speed of re-entrainment based on the calculation of wheel running activity. Furthermore, TRF mice were even healthier than those that were not given a shift or a TRF. This suggests that although mice may not be fully recovered one week after a shift [3,17], the positive effects of TRF outweigh the adverse outcomes of a mild disruption to the circadian rhythm. These results imply that health status does not necessarily correlate with behavioral adaptation and raise a possibility that any nighttime TRF, if not too short, is protective in mice under day–night reversal conditions. To test this hypothesis, we subsequently applied whole night TRF to mice before, after, or both before and after a 12-h shift. The results of locomotor activity showed that the group treated with the whole night TRF after the shift was the slowest group for re-entrainment. However, this group and the group treated with TRF both before and after shift were much healthier than ad libitum controls, whereas TRF before shift only had no such effect. It indicates that TRFs in progress, as long as it is not passed when LPS is applied, offer solid protection, which echoes a number of reports of benefits of TRF during the active phase [5,14,15,18,19,20,21].

Although both 6-h and 12-h TRFs were protective in our study, different TRF strategies may differ in many aspects, including clock oscillation, energy metabolism, health, and lifespan [5,22,23]. For example, human subjects with prediabetes treated with 6-h TRF experienced greater improvement in insulin sensitivity, blood pressure, and oxidative stress than those with 12-h TRF [24]. At the same time, differences under circadian disruptions need further studies.

Despite a positive correlation between adjustment speed and health status in our previous report [3], all three experiments in the current study revealed that this is not always the case. Therefore, the research on adaptation cannot simply rely on behavioral experiments. It is worthwhile to monitor parameters in real-time that can provide insight into the oscillation of internal clocks, such as blood pressure and heart rate [25,26]. Nevertheless, evaluation of health status is the most important and should not be ignored. Since a single phase shift is too mild to cause obvious health problems in normal mice, we used a sepsis model to evaluate the health status [3,17]. In addition, there are good correlations between the septic scores and wheel revolutions, suggesting that the health status of septic mice can be reflected by locomotor activities. This approach may provide a better way to assess health status by avoiding the subjectivity and uncertainty of double-blind experiments. Nevertheless, investigation of mild disease models, rather than a lethal model, and screening for biomarkers that are sensitive to jet lag could be future directions.

In summary, by studying behavior and health status in mice, we revealed the beneficial effect of TRF strategies in certain circumstances of phase shifts. Even though behavioral fitness does not correlate well with health status, no TRF strategy that we applied deteriorates LPS-induced sepsis. Hence, strictly eating during the active phase may always be a good choice, even for those suffering from jet lag. Given that TRFs will offer great benefits to health, which is exceptionally important for the sick under the condition of circadian disruption. Future studies may focus on the long-term effect of TRF on chronic jet lag. The underlying molecular mechanisms need to be investigated as well.

## 5. Conclusions

We revealed the beneficial effects of given TRF strategies in certain phase-shifting situations. Although behavioral fitness did not correlate well with health status, none of the TRF strategies we used deteriorated lipopolysaccharide-induced sepsis, suggesting that TRFs during the dark phase may offer great health benefits, especially for the sick under circadian disruption.

## Figures and Tables

**Table 1:** Sepsis score.

Schedule	Hours after LPS	Type of Sepsis Score	Group o	Group a	Group b	Group c	Group d	Group e	Other Comparisons (Low vs. High, *p* < 0.05)
Experiment 1 (6-h delay shift + 6-h TRFs)	3	Level of consciousness	-	0.43 ± 0.14	0.50 ± 0.14	0.50 ± 0.13	0.43 ± 0.14	0.07 ± 0.07	-
		Response to stimuli	-	0.50 ± 0.14	0.21 ± 0.11	0.21 ± 0.11	0.07 ± 0.07	0.14 ± 0.10	-
	9	Level of consciousness	-	1.79 ± 0.11	1.21 ± 0.15	1.21 ± 0.19	0.57 ± 0.14 ***	0.79 ± 0.15 ***	d vs. b, c
		Response to stimuli	-	1.43 ± 0.14	1.00 ± 0.15	1.00 ± 0.18	0.50 ± 0.17 **	0.64 ± 0.17 **	-
	15	Level of consciousness	-	2.36 ± 0.13	2.14 ± 0.14	1.79 ± 0.11 *	1.64 ± 0.13 **	1.64 ± 0.17 **	-
		Response to stimuli	-	2.36 ± 0.13	2.14 ± 0.14	1.79 ± 0.11 *	1.64 ± 0.13 **	1.21 ± 0.19 ***	e vs. b, c
Experiment 2 (12-h shift + 6-h TRFs)	3	Level of consciousness	0.29 ± 0.13	0.50 ± 0.14	0.69 ± 0.13	0.36 ± 0.13	0.71 ± 0.13	0.43 ± 0.14	-
		Response to stimuli	0.14 ± 0.10	0.14 ± 0.10	0.08 ± 0.08	0.07 ± 0.07	0.14 ± 0.10	0.07 ± 0.07	-
	9	Level of consciousness	2.07 ± 0.13	1.79 ± 0.11	1.39 ± 0.14	1.21 ± 0.11 *	1.64 ± 0.13	1.21 ± 0.11 *	b, c, e vs. o
		Response to stimuli	1.93 ± 0.07	1.93 ± 0.07	1.39 ± 0.14 *	1.29 ± 0.13 **	1.29 ± 0.13 **	1.21 ± 0.11 ***	b, c, d, e vs. o
	15	Level of consciousness	2.21 ± 0.19 *	2.79 ± 0.11	1.92 ± 0.14 ***	1.21 ± 0.11 ***	1.86 ± 0.10 ***	1.50 ± 0.14 ***	b, c, d, e vs. o
		Response to stimuli	2.36 ± 0.13	2.79 ± 0.11	1.46 ± 0.18 ***	1.29 ± 0.12 ***	1.86 ± 0.10 ***	1.43 ± 0.14 ***	b, c, d, e vs. o
Experiment 3 (12-h shift + 12-h TRFs)	3	Level of consciousness	-	0.36 ± 0.13	0.29 ± 0.13	0.38 ± 0.14	0.21 ± 0.11	-	-
		Response to stimuli	-	0.36 ± 0.13	0.21 ± 0.11	0.54 ± 0.14	0.43 ± 0.14	-	-
	9	Level of consciousness	-	2.21 ± 0.24	2.29 ± 0.16	1.62 ± 0.18	1.50 ± 0.27	-	-
		Response to stimuli	-	2.07 ± 0.25	2.07 ± 0.22	1.39 ± 0.18	1.64 ± 0.27	-	-
	15	Level of consciousness	-	2.71 ± 0.24	2.86 ± 0.10	2.15 ± 0.15	1.79 ± 0.32 *	-	d vs. b
		Response to stimuli	-	2.71 ± 0.22	2.71 ± 0.13	2.15 ± 0.10	1.71 ± 0.27 **	-	d vs. b

*** *p* < 0.001; ** *p* < 0.01; * *p* < 0.05, vs. group a, One-way ANOVA with Tukey’s multiple comparisons test.

## Data Availability

Not applicable.
